# Molecular Mechanisms of Intervertebral Disc Degeneration Induced by *Propionibacterium acnes*

**DOI:** 10.1155/bmri/5513856

**Published:** 2025-04-15

**Authors:** Weichao Yang, Yude Xu, Yong Tan, Jinzhi Lin, Huan Chen, Shaojin Li, Haixiong Miao, Dongping Ye

**Affiliations:** ^1^Guangzhou Red Cross Hospital, Guangzhou Red Cross Hospital of Jinan University, Guangzhou, China; ^2^Department of Pain Medicine, Guangzhou Red Cross Hospital, Guangzhou Red Cross Hospital of Jinan University, Guangzhou, China

**Keywords:** inflammation, intervertebral disc degeneration, molecular mechanism, *Propionibacterium acnes*, pyroptosis

## Abstract

Intervertebral disc degeneration (IVDD), a prevalent degenerative disorder with substantial socioeconomic impacts, is closely linked to endplate inflammation and chronic low back pain. Its pathogenesis involves multifactorial mechanisms, including long-term chronic mechanical loading, external trauma, and hereditary factors. Emerging evidence highlights *Propionibacterium acnes* (*P. acnes*), a gram-positive bacterium with potent proinflammatory properties, as a key contributor to IVDD progression. This review systematically analyses the latest literature on related studies, focusing on the molecular mechanisms of IVDD induced by *P. acnes*. Three molecules play an important role in the induction of IVDD by *P. acnes*, namely, IL-1*β*, MIF, and MMP. In addition, *P. acnes* induces IVDD through three core mechanisms, namely, proinflammatory (activation of TLR2, production of large amounts of ROS to promote inflammation), pyroptosis (production of large amounts of NLRP3 through the TXNIP-NLRP3 axis and the ROS-NLRP3 axis), and apoptosis (promotion of Bax and inhibition of Bcl-2 expression through the TLR2-JNK pathway). The dissection of these related important molecules and pathogenic mechanisms can lead to a better understanding of the role of *P. acnes* in IVDD. It can provide an important theoretical basis for future research. However, the current study's lack of large-scale clinical validation, unresolved colonization controversies, and limited experimental methods are limitations. Therefore, in the future, it is still necessary to improve the relevant theories and resolve the current controversies through more advanced experimental methods and higher quality clinical studies. In conclusion, the study of *P. acnes*–induced IVDD is promising, and further research can be conducted in the future, which is expected to develop novel therapeutic approaches for *P. acnes*, thus effectively slowing down the development of IVDD.

## 1. Introduction

Intervertebral disc degeneration (IVDD) is one of the common degenerative diseases in spinal surgery, which can lead to spinal stenosis, vertebral instability, low back pain, and spinal deformity [1]. IVDD not only affects patients' daily work and quality of life, but it also raises the economic burden on both patients and society [2]. However, the pathogenic mechanisms of IVDD are complex, and the precise pathophysiological processes are not entirely understood. Currently, long-term chronic mechanical loading, external trauma, and hereditary factors are considered potential risk factors for IVDD [3].

*Propionibacterium acnes* (*P. acnes*) is a microaerophilic, anaerobic, aerotolerant gram-positive rod-shaped bacterium that lives naturally in the human skin, oral cavity, conjunctiva, stomach, and external auditory canal [4]. It can cause several diseases, such as endocarditis, prostatitis, and sarcoidosis. Stirling et al. identified *P. acnes* in degenerative intervertebral discs with a positive rate of 44.4% (16/36) [5]. Other studies have now established that *P. acnes* colonizes degenerative discs, with favourable rates ranging from 13.5% to 32.8% [6–9]. In contrast, this bacteria was nearly entirely missing from normal intervertebral discs.

Several animal studies have confirmed that *P. acnes* plays an important role in developing IVDD. In a study by Zamora et al., it was discovered that injecting *P. acnes* into rats' intervertebral discs hastened the progression of IVDD [10]. Chen et al. discovered that infection of normal rabbits with *P. acnes* caused severe IVDD and Modic change (MC) [11]. Shan et al. discovered that injecting *P. acnes* into the lumbar intervertebral discs of healthy rabbits exacerbated IVDD and degeneration in a time-dependent manner [12].

However, most studies on *P. acnes* have concentrated on bacterial detection methods and rates rather than the molecular mechanisms underlying bacterial-induced IVDD. As a result, this review summarized the literature on *P. acnes*–induced IVDD and explored its key effector molecules and pathogenic mechanisms. Finally, the paper addresses the limits of previous studies and considers future research prospects.

## 2. *P. acnes* and Its Pathogenic Properties

*P. acnes* is also known as *Cutibacterium acnes* [13]. It can persist for up to 8 months in anaerobic conditions in vitro, making it a stringent anaerobe [14]. Interestingly, Cove et al. discovered that *P. acnes* can also grow at high oxygen concentrations but have a short survival time [15]. It shows that the bacterium is highly environmentally adaptable and can survive in various conditions. *P. acnes* divides slowly, once every 5.1 h [4]. It means that the bacterium cannot overgrow for short periods, resulting in acute, explosive infections. Furthermore, it is highly resistant to phagocytosis and persists by evading macrophage phagocytosis and remaining latent within them [16].

There are many types of *P. acnes*. Johnson et al. divided *P. acnes* into Types I and II based on agglutination tests and cell wall polysaccharides [17]. McDowell et al. then used recA gene sequence analysis to identify a distinct bacterial species from the prior *P. acnes* Types I and II in disc samples, which they called Type III [18].

**Figure 1:**
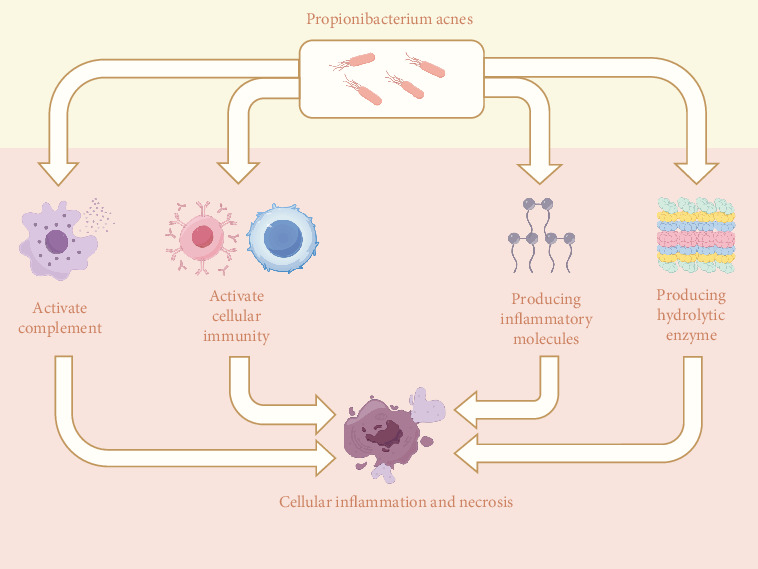
The primary pathogenic mechanism for *P. acnes*. *P. acnes* can produce cellular inflammation and necrosis mostly by activating complement, stimulating cellular immunity, and generating inflammatory factors and hydrolytic enzymes.

*P. acnes* is very proinflammatory and has multiple pathogenic pathways (Figure 1). First, *P. acnes* causes inflammation by producing several hydrolytic enzymes. Hoeffler et al. discovered that *P. acnes* synthesizes hydrolytic enzymes such as hyaluronidase, deoxyribonuclease, and phosphatase and that it was also the most active bacterial type in synthesizing hydrolytic enzymes among the bacteria studied [19]. Second, *P. acnes* can cause inflammation by releasing proinflammatory chemicals. Allaker et al. discovered that *P. acnes* generates histamine, tryptamine, and short-chain fatty acids that cause inflammation and pain [20]. Third, *P. acnes* can cause inflammation by activating complement. Webster et al. discovered that *P. acnes* can activate complement and stimulate C5-dependent chemokine synthesis, resulting in an inflammatory response [21]. Finally, *P. acnes* can cause inflammation by affecting the body's immune system. Ishimura et al. discovered that *P. acnes* may have a proinflammatory effect by activating humoral and cellular immunity, resulting in local tissue damage [22].

*P. acnes* reproduces slowly, yet it may thrive in various environments and resist phagocytosis, which explains its low virulence and long-lasting infectious properties. At the same time, *P. acnes* secretes several hydrolytic enzymes and activates the immune system, causing inflammation and tissue damage. These traits establish *P. acnes*' propensity to induce various inflammatory disorders, particularly chronic inflammatory diseases.

## 3. The Association Between *P. acnes* and IVDD

### 3.1. Findings of *P. acnes* in Intervertebral Discs and Its Clinical Significance

Stirling et al. discovered *P. acnes* colonization in degenerative intervertebral discs for the first time in 2001 [5]. They collected disc tissues from 36 patients for culture and 19 tested positive for bacteria on culture. *P. acnes* was found in 16 of the 19 positive samples. They also cultivated disc tissue from individuals with various spinal problems, such as scoliosis, fractures, and myeloma, to exclude the possibility of chance in the results. Surprisingly, their cultures were all negative. This finding revealed that *P. acnes* plays an essential role in the development of IVDD, giving rise to the disc infection theory. This pioneering finding has been repeated in later investigations. For example, Albert et al. found 26 positive cultures of *P. acnes* in disc tissue from 61 individuals, with only two considered contaminated by sampling since two different bacteria were cultured [6]. Zhou et al. used polymerase chain reaction (PCR) to detect *P. acnes*–specific 16S rDNA sequences in disc tissue from 46 patients [7]. The investigation found that 12 samples were positive, including nine positive disc tissues, one positive paravertebral muscle, and two double-positive samples.

*P. acnes* is not the only microorganism discovered in degenerative discs; others include coagulase-negative staphylococci, *Corynebacterium* spp., and alpha-hemolytic streptococci. However, some research has confirmed that *P. acnes* may be the primary cause of IVDD. In a prospective observational study, Senker et al. discovered that *P. acnes* was the most prevalent pathogen in degenerative discs with positive bacterial cultures [23]. In an observational study, Georgy et al. discovered that *P. acnes* was frequent in degenerative cervical discs [24]. In a comprehensive review and meta-analysis study, Ganko et al. discovered that individuals with symptomatic IVDD had a greater prevalence of infections than asymptomatic patients, with *P. acnes* being the most prevalent bacterial species to cause infections [25].  Table 1 shows the results of positive culture tests for *P. acnes* in IVDD patients and other relevant data.

Because the blood vessels in the annulus fibrosus have entirely deteriorated by adulthood, the disc has no communication with the external circulation other than the diffusion of nutrients through the endplates, and it has always been considered a closed and sterile tissue. However, identifying *P. acnes* in degenerating discs has provided new insights into the therapeutic possibilities for IVDD, particularly antibiotics. Albert et al. found that oral amoxicillin–clavulanic acid considerably alleviated IVDD-induced lower back discomfort [26]. Another randomized controlled trial (RCT) investigation confirmed this finding [27]. It is worth noting that the medication in that trial was used for up to 90 days, which raised the chance of infection with drug-resistant bacteria. The study's high medication dose may have been due to the low diffusion ability of *β*-lactams in bone and disc tissues [28]. In contrast, rifampicin appears to be more effective in treating *P. acnes* [29]. Rifampicin has a low minimum inhibitory concentration (MIC) against *P. acnes*, indicating that the organism is very susceptible. Rifampicin is more effective at diffusing into bone and disc tissue than *β*-lactam medicines [28]. Rifampicin should be used with other antimicrobials to limit the danger of resistance [30]. In vivo, rifampicin and *β*-lactam antimicrobials have a cure rate of 36% and 4%, respectively, but when taken together, the cure rate increases to 63% [29]. Combining rifampicin with *β*-lactams may improve antibacterial activity, although further clinical confirmation is needed.

### 3.2. The Controversy of *P. acnes* Colonization in Intervertebral Discs

Because *P. acnes* can be found in various locations in the human skin, oral cavity, and external auditory canal, some researchers speculate that the bacterium was introduced into the disc tissue by accident during surgical puncture or sample collection rather than from within the tissue [31]. Carricajo et al. found a positive culture of *P. acnes* in only two cases of disc tissue from 54 patients and a positive bacterial culture of ligamentum flavum and muscle tissue in these two cases. They concluded that the bacteria in the disc was due to surgical contamination [32]. Ben-Galim et al. found only two positive bacterial cultures in disc tissue from 30 patients, and the bacteria were coagulase-negative staphylococci rather than *P. acnes*, implying that the positive cultures were due to contamination and that there was no evidence of an association between the bacteria and the disease [33]. However, it is important to note that certain factors influenced the findings of this research. First, Carricajo et al.'s study clearly expressed a difference in sampling duration between ligamentum flavum and muscle tissue, but their results did not reflect this distinction and combined both. It could significantly boost the positivity of bacterial cultures in these tissues, influencing the outcome. Second, in the study by Ben-Galim et al., patients were explicitly advised that preventive antibiotics were not stopped before surgery, which could explain the low rate of positive bacterial cultures.

It is undeniable that the contamination factor in that controversy will more or less impact the results, as current presurgical skin preparation treatments do not appear capable of entirely killing *P. acnes* [34, 35]. Savage et al. found that surgical sterilizing reduced the prevalence of *P. acnes* infection on the skin from 84% before to 6% after sterilization. However, it was not a total eradication [34]. However, it would be unwise to credit all outcomes to contamination, as there is ample evidence that *P. acnes* originates from degenerating discs. As previously stated, Zhou et al. [7] conducted similar tests to Carricajo et al.'s [32], although the former results appear more reasonable. Zhou et al. only removed muscle tissue as a comparative verification, significantly lowering the probability of false-positive results. Second, their sampling of muscle tissue occurred intraoperatively, at a much shorter interval than disc tissue sampling, resulting in fewer discrepancies. Finally, they used a more reliable PCR approach for validation than simple bacterial cultures, resulting in better legitimacy of the findings. Most significantly, they did not entirely refute the effect of contamination on the results but instead used proportions to demonstrate that positive bacterial cultures came from within the degenerating discs, with contamination accounting for just a tiny fraction of the outcome. In addition, McDowell et al. confirmed that the type of *P. acnes* in the degenerated discs was mostly Type III rather than Types I and II, which are widely colonized in areas such as the skin and the oral cavity, indicating that it does not all originate from contamination [18].

### 3.3. The Hypothesis of Invasion of the Intervertebral Disc by *P. acnes*

There are currently two hypotheses regarding how *P. acnes* invades the disc. The first hypothesis is that the bacteria may enter through a breach in the annulus fibrosus. According to Stirling et al., a slight injury ruptures the integrity of the disc's annulus fibrosus, allowing *P. acnes* to invade and cause an inflammatory response that leads to IVDD [5]. Capoor et al. studied 119 cases of culture-positive disc tissues of *P. acnes* from 368 patients, and the majority of the 119 cases were prolapsed and free disc herniation types, which were accompanied with significant annulus fibrosus rupture [36]. This behaviour is also seen in a study by Zhou et al. [7]. All nine culture-positive disc samples showed severe fibrous ring rupture. Another hypothesis is that the bacteria could enter the disc via haematogenous dissemination. *P. acnes* widely colonizes in the oral cavity and mucous membranes; if these places burst, there is a high risk of infection entering the bloodstream. Although the intervertebral disc is not vascularised, material exchange occurs through the endplates rich in blood vessels. Hence, blood-borne bacteria are likely to reach the disc through this area. MC, as an imaging manifestation, may help to diagnose endplate inflammation. Albert et al. discovered that a higher culture positivity for *P. acnes* was found in disc tissues incorporating MC [6]. An animal experiment indicated that after inoculating rabbits with *P. acnes*, most developed MC in addition to IVDD [11]. These findings not only support this form of bacterial invasion but also help explain why MC is more likely to occur after bacterial infection.

## 4. Key Molecules Involved in the Induction of IVDD by *P. acnes*

### 4.1. Interleukin-1*β* (IL-1*β*)

The IL-1 family, particularly IL-1*β*, plays a crucial role in developing IVDD [37]. One study indicated that IL-1*β* was considerably higher in degenerative disc tissue than in normal disc tissue [38]. Meanwhile, positive feedback regulation between IL-1*β* and IL-6 enhances the local inflammatory response, speeding up the development of IVDD [39]. One of the distinguishing characteristics of IVDD is the disturbance of the extracellular matrix (ECM). It found that the inhibition of IL-1*β* can reduce ECM disruption and ease IVDD development [40]. In addition, Purmessur et al. discovered that IL-1*β* increased the expression of NGF and BDNF in the intervertebral discs, explaining the incidence of IVDD-associated low back pain [41].

*P. acnes* infection increases inflammatory cytokines such as TNF-*α*, IL-6, IL-8, and IL-1*β* levels in tissues [42]. Kistowska et al. reported elevated levels of IL-1*β* in tissues infected with *P. acnes* [43]. Capoor et al. infected normal intervertebral disc cells with *P. acnes* and investigated its ability to cause IVDD by measuring changes in inflammatory and neurotrophic factors [44]. The researchers discovered that IL-1*β* expression peaked at a multiplicity of infection (MOI) of 1:1000. Also, they confirmed the association of IL-1*β* expression with the expression of IL-6, CCL3, and CCL4. It shows that IL-1*β* acts as both an inflammatory effector and a modulator. In conclusion, IL-1*β* plays a key role in the induction of IVDD by *P. acnes*.

### 4.2. Macrophage Inhibition Factor (MIF)

MIF is classified as a pleiotropic cytokine that affects various immune cells [45]. It has direct proinflammatory effects in various inflammatory illnesses, including sepsis, rheumatoid arthritis, and glomerulonephritis [46]. Among them, MIF can stimulate inflammatory responses by interacting with CD74, thereby exacerbating the degeneration of cartilage endplate tissue and inducing IVDD [47]. Zhang et al. discovered that MIF expression was more significant in human degenerated cartilage endplate samples [48]. Furthermore, they discovered that MIF expression increased following *P. acnes* injection into rat intervertebral discs, resulting in IVDD. To examine how *P. acnes* regulates MIF at the molecular level, they used western blotting and qPCR to detect MIF expression at the protein and mRNA levels. The study's findings revealed that MIF expression was not significantly affected at the mRNA level but was elevated at the protein level, implying that *P. acnes* regulates MIF primarily at the translational stage. The study found that NF-*κ*B inhibitors dramatically lowered MIF expression, indicating that *P. acnes* may increase MIF expression via the NF-*κ*B pathway. Therefore, MIF may be one of the key molecules involved in the induction of IVDD by *P. acnes*.

### 4.3. Matrix Metalloproteinase (MMP) Family

The MMP family regulates ECM remodelling and inflammatory processes [49]. Meanwhile, MMP-mediated ECM degradation plays a significant role in the progression of IVDD [50]. In degenerated disc tissues, the activity and expression of several types of MMPs are increased [51]. Choi et al. discovered that *P. acnes* increased MMP2 expression via TNF-*α* [52]. Jugeau et al. discovered that *P. acnes* boosted the production and release of MMP9 via the TLR signalling pathway [53]. As a result, MMPs that are released during *P. acnes* stimulation may play a significant role in causing IVDD.

Lan et al. discovered that stimulation with various forms of *P. acnes* causes intervertebral disc tissue to release distinct types of MMP family proteins [54]. *P. acnes* Types I and II increased the expression of MMP13, while Type III raised the expression of MMP3. Similarly, Zhang et al. discovered higher MMP13 levels in *P. acnes*–induced degenerative intervertebral discs [21]. Furthermore, Zheng et al. discovered that intervertebral discs treated with *P. acnes* increased MMP1 expression while decreasing tissue inhibitor of metalloproteinase-1 (TIMP1) expression [55]. Thus, in addition to direct stimulation, *P. acnes* controls MMP expression by influencing MMP inhibitors.

## 5. Key Mechanisms Involved in the Induction of IVDD by *P. acnes*

### 5.1. *P. acnes* Induces IVDD Through the Proinflammatory Mechanism

As previously noted, *P. acnes* can cause inflammation in various ways. Hence, inducing IVDD through inflammation is a significant factor, with the TLR signalling pathway playing a key role. The TLR signalling system is an important mechanism in regulating innate immunity, and it can be activated by a range of external stimuli, including almost all microbial infections [56]. TLR2, a key member of the toll-like receptor family, stimulates the NF-*κ*B pathway, causing inflammation and apoptosis [57]. Su et al. discovered that TLR2 recognition may play an important role in the interaction between *P. acnes* and its host [58]. Jiao et al. discovered that disc tissues implanted with *P. acnes* significantly expressed inflammatory markers, precisely IL-8 [59]. They discovered that reducing TLR2 and p65 expression improved the condition, which confirmed the unique regulatory link.

Reactive oxygen species (ROS) are unstable and highly reactive molecules that are typically produced during oxygen metabolism in the body, such as superoxide anion (O_2_^−^) and hydrogen peroxide (H_2_O_2_) [60]. ROS serve critical regulatory roles in physiological systems like oxidative, immunological, and proinflammatory responses [61]. Tsai et al. discovered that *P. acnes* stimulated iNOS/NO and COX-2/PGE2 synthesis in several cells by activating ROS-dependent pathways [62]. Previously, investigations have demonstrated that active iNOS and COX-2 can enhance NO and PGE2 generation in response to disc damage or noxious stimuli, resulting in inflammation and IVDD [63, 64]. Lin et al. conducted in vivo and in vitro experiments and replicated the findings [65]. *P. acnes* promotes inflammation by activating the NF-*κ*B pathway and producing high levels of ROS [65].

The above findings not only show that proinflammatory mechanisms are significant in the production of IVDD by *P. acnes*, but they also explain why IVDD patients with severe lower back pain symptoms are more likely to have positive bacterial cultures. *P. acnes* causes disc tissue to produce more molecules, such as IL-8, COX-2, and PGE2, which cause pain [66]. Future investigations on *P. acnes*' proinflammatory processes will focus on the NF-*κ*B pathway, which appears to have a role in both the exogenous activation of the TLR2 proinflammatory process and the endogenous creation of the ROS proinflammatory process.

### 5.2. *P. acnes* Induces IVDD Through the Pyroptosis Mechanism

Pyroptosis is a novel type of programmed inflammatory cell death characterized by cell swelling, membrane rupture, and release of proinflammatory cytokines [67]. Among these, the gasdermin (GSDM) family and the inflammatory complexes play important roles in this process. GSDMs primarily mediate the process of cell membrane rupture during pyroptosis, and gasdermin D (GSDMD) is the primary effector molecule [68]. As a result, GSDMD is thought to play a critical role in pyroptosis [69]. Zhang et al. created a mouse model of IVDD utilizing fine-needle puncture of the intervertebral disc and discovered that the expression of GSDMD in the model group was significantly higher than in the sham operation group [70]. Zhou et al. successfully reversed IVDD by inhibiting GSDMD using multifunctional metal polyphenol nanoparticles [71]. Liao et al. discovered that enhancing cellular autophagy to reduce GSDMD accumulation in cells might successfully prevent nucleus pulposus cell pyroptosis and delay IVDD [72]. Inflammatory complexes primarily promote inflammation during pyroptosis, with NLRP3 as the primary effector molecule [73]. Tang et al. found that inhibiting NLRP3 could somewhat delay IVDD [74]. Wang et al. reported the same outcome [75]. As a result, the suppression of NLRP3 is investigated as a potential method for reducing the inflammatory response and rescuing IVDD.

TXNIP was discovered as a possible NLRP3 regulator [76]. TXNIP is an endogenous thioredoxin inhibitor, and the overexpression of this protein causes increased ROS generation and cellular oxidative stress [77]. He et al. discovered that NLRP3, TXNIP, IL-1*β*, and IL-18 expressions and secretion levels rose significantly following disc tissue infection with *P. acnes* [77]. Inhibiting TXNIP expression using siRNA led to reduced levels of pyroptosis-related molecules like NLRP3, IL-1*β*, and caspase-1. It shows that *P. acnes* can trigger pyroptosis via the TXNIP-NLRP3 axis, accelerating IVDD.

In addition to being proinflammatory, ROS can trigger cellular pyroptosis [78]. Ma et al. discovered that ROS can increase the expression of NLRP3, causing pyroptosis in nucleus pulposus cells [79]. Zhao et al. discovered ROS could activate the NF-*κ*B signalling pathway, resulting in NLRP3 overexpression and nucleus pulposus cell pyroptosis [80]. As a result, the creation of NLRP3 and ROS appear to be inextricably linked. Tang et al. cocultured intervertebral disc cells with *P. acnes* and discovered enhanced expression of ROS and NLRP3 in the disc cells [81]. To illustrate the link between ROS and NLRP3, they found that the inhibition of ROS led to downregulation of NLRP3, but the inhibition of NLRP3 only reduced the expression of downstream pyroptosis-associated proteins and the proportion of cells undergoing pyroptosis. It suggests that *P. acnes* can cause IVDD by inducing nucleus pulposus cell pyroptosis via the ROS-NLRP3 pathway.

In the process of inducing IVDD by *P. acnes* by pyroptosis, NLRP3 is more significant than GSDM. The authors believe that there may be the following reasons for this phenomenon. NLRP3 primarily promotes inflammation during the pyroptosis process, consistent with *P. acnes*' pathogenic features. Furthermore, *P. acnes* produces several proinflammatory chemicals that facilitate the synthesis of NLRP3. It is worth noting that GSDM, an essential effector molecule of cell killing during pyroptosis, appears to be overlooked in the preceding investigations. However, blocking the expression of GSDM can effectively reduce the frequency of pyroptosis. As a result, further research into the association between *P. acnes* and GSDM is required, which will aid in discovering new treatment targets.

### 5.3. *P. acnes* Induces IVDD Through the Apoptosis Mechanism

Apoptosis is the most prevalent type of programmed cell death, and it controls cellular processes in development, homeostasis, and disease. Previous research has demonstrated that apoptosis plays an important role in the pathophysiology of IVDD. Wang et al. created a rabbit model of IVDD and discovered that the average apoptotic index in degenerated discs was significantly greater than in normal discs [82]. Park and colleagues discovered that the expression of apoptosis-related markers such as caspase-9, caspase-3, and cytochrome-c was considerably enhanced in degenerative disc tissue [83].

Bax and Bcl-2 are regulatory proteins that play a key role in regulating apoptosis. Bax and Bcl-2 are members of the Bcl-2 protein family, which produces hetero- or homodimers that can function as anti- or proapoptotic regulators in various cellular processes [84]. Bax has an important function in mitochondrial apoptosis. It can undergo structural changes when stressed, resulting in apoptosis [85–87]. In contrast, Bcl-2 suppresses apoptosis in various biological systems [88]. Thus, the ratio of Bax to Bcl-2 determines whether cells survive or die during apoptosis.

Lin et al. conducted important in vivo and in vitro studies [89]. The in vivo experiments were performed by injecting *P. acnes* into the intervertebral discs of mice for infection. In vitro experiments were conducted by coculturing the bacteria with nucleus pulposus cells to simulate the infection state. They discovered that Bcl-2 protein expression was downregulated, while Bax protein expression was upregulated, indicating that *P. acnes* may increase apoptosis and speed up the process of IVDD. Additionally, they discovered that *P. acnes* might activate the apoptotic process via TLR2. TLR2 regulates several downstream pathways, although the JNK pathway was the most important in this process. This finding was also confirmed in another investigation [90].

Several apoptotic pathways highly associated with IVDD have been identified, such as the Wnt/*β*-catenin signalling pathway, PI3K/AKT signalling pathway, and mTOR signalling pathway [91–93]. Unfortunately, compared to proinflammatory and pyroptosis, there are fewer investigations on *P. acnes*–induced IVDD via apoptosis. Therefore, this research question still needs to be supplemented with more high-quality findings in the future.

## 6. Conclusion

IVDD is one of the leading causes of persistent low back pain and motor dysfunction, affecting patients' quality of life and work productivity while also imposing a significant economic burden on society. As research has progressed, the potential pathogenic role of *P. acnes* in IVDD has gradually been revealed. This finding not only provides a new perspective for understanding the complex pathophysiological process of IVDD, but it may also provide a theoretical basis for developing new therapeutic approaches.

**Figure 2:**
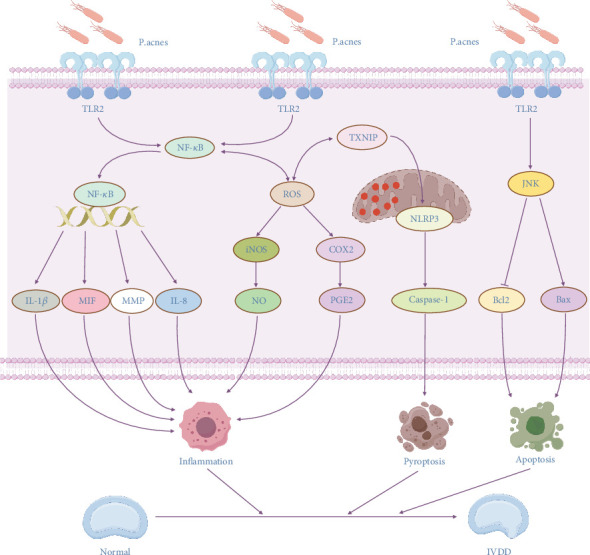
Schematic diagram of the network of molecular mechanisms of IVDD induced by *P. acnes*.

This review focuses on *P. acnes*' role in IVDD and the molecular mechanisms behind it. This review summarizes previous relevant studies to reveal the multifaceted roles of *P. acnes* in IVDD, and these mechanisms are interconnected and together form a complex network of *P. acnes*–induced IVDD (Figure 2). First, this study summarizes the key chemicals implicated in *P. acnes*–induced IVDD. IL-1*β* and MIF have proinflammatory actions that can worsen local inflammation and increase the risk of IVDD. The MMP family can disrupt the ECM of the intervertebral disc through its degradation, thus accelerating the development of IVDD. This review also focuses on three important mechanisms by which *P. acnes* induces IVDD. The proinflammatory mechanism by activating TLR2 and the production of ROS to induce an inflammatory response, to release inflammatory factors, result in IVDD. The pyroptosis mechanism promotes NLRP3 expression in cells via the TXNIP-NLRP3 axis and the ROS-NLRP3 pathway, resulting in cell death and the release of proinflammatory molecules, worsening the severity of IVDD. The apoptotic mechanism activates Bax expression and inhibits Bcl-2 expression via the TLR2-JNK pathway axis, promoting cell death and hastening the development of IVDD. The identification of these pathways not only opens up new therapy options for IVDD but also contributes to a better understanding of *P. acnes*' role in other chronic inflammatory disorders. Furthermore, the identification of *P. acnes* in IVDD has significant implications for treatment. Antibiotic medication, for example, may emerge as a new technique for treating IVDD symptoms, but the current findings are contentious and require further clinical validation.

Although significant progress has been made in past investigations on the induction of IVDD by *P. acnes*, certain limitations remain. First, most existing studies are based on animal and in vitro cellular tests, with no large-scale clinical confirmation. More high-quality clinical investigations are needed in the future to confirm *P. acnes*' pathogenic role in IVDD and its therapeutic potential. Second, the debate concerning *P. acnes* colonization in IVDD has yet to be settled, and more reliable assays must be developed to discriminate between sample contamination and actual pathogen colonization. In addition, current studies have focused on the proinflammatory, pyroptosis, and apoptosis mechanisms of *P. acnes*, and other potential mechanisms (e.g., metabolic regulation and ferroptosis) of *P. acnes* in IVDD are still less investigated. Future studies should further explore these mechanisms to understand the full role of *P. acnes* in IVDD.

In conclusion, this review summarizes the molecular mechanisms of *P. acnes* in producing IVDD using available findings, providing an up-to-date theoretical perspective for future related research and clinical therapy. Further research is likely to lead to the development of novel therapeutic techniques for *P. acnes*, thereby slowing the progression of IVDD.

## Figures and Tables

**Table 1:** A review of experimental information on a positive culture of *Propionibacterium acnes* in intervertebral disc tissue.

**Experimental specimens**	**Subject age**	**Bacterial culture environment**	**Incubation time**	**Detected positive rate**	**References**
Cervical disc	Mean age 55 years, age range 18–87 years	Aerobic conditions, 5% CO_2_	7 days	14/48 (29%)	[24]
Lumbar disc	Mean age 43.9 years	Anaerobic conditions, 5% CO_2_, 35°C	5 days	7/52 (13.5%)	[8]
Lumbar disc	Mean age 54.7 years, range 22–75 years	Anaerobic conditions, 10% CO_2_, 37°C	14 days	11/46 (23.9%)	[7]
Lumbar disc	Mean age 46.4 years	Anaerobic conditions, 37°C	7 days	26/61 (43%)	[6]
Cervical disc, thoracic disc, lumbar disc	Mean age 58.09 years	Anaerobic conditions, 5% CO_2_, 36°C	7 days	74/410 (18.05%)	[23]
Lumbar disc	Mean age 55.30 ± 14.59 years	Anaerobic conditions, 10% CO_2_, 37°C	14 days	20/76 (26.32%)	[9]
Lumbar disc	—	Anaerobic conditions, 37°C	7 days	16/36 (44.5%)	[5]

## Data Availability

Data sharing is not applicable to this article as no datasets were generated or analysed during the current study.

## Nomenclature

IVDD: intervertebral disc degeneration
*P. acnes*: *Propionibacterium acnes*
MC: Modic change
PCR: polymerase chain reaction
RCT: randomized controlled trial
MIC: minimum inhibitory concentration
IL-1*β*: interleukin-1*β*
ECM: extracellular matrix
MOI: multiplicity of infection
MIF: macrophage inhibition factor
MMP: matrix metalloproteinase
TIMP1: tissue inhibitor of metalloproteinase-1
ROS: reactive oxygen species
GSDM: the gasdermin family
GSDMD: gasdermin D
